# Supplementary material for: Effectiveness and Safety of Ayurvedic Medicines in Type 2 Diabetes Mellitus Management: A Systematic Review and Meta-Analysis
Source: Front Pharmacol. 2022 Jun 8;13:821810. doi: 10.3389/fphar.2022.821810 (PMC9213670; doi:10.3389/fphar.2022.821810)
Supplement: Supplementary file 1 [file DataSheet1.zip › Supplementary Material/Forest and Funnel Plots/Zingiber officinale/Waist circumference.pdf]

Zingiber officinale - Waist circumference

Study	Intervention N	Intervention Mean	Intervention SD	Comparator N	Comparator Mean	Comparator SD	Mean Diff. with 95% CI	Weight (%)
Arablou 2014a, Arablou 2014b	33	93.60	9.46	30	94.10	8.41	-0.50 [-4.94, 3.94]	27.21
Azimi 2014, Azimi 2016	41	104.17	8.32	39	100.66	8.74	3.51 [-0.23, 7.25]	36.84
Shidfar 2015	22	97.90	7.90	23	96.90	10.10	1.00 [-4.31, 6.31]	19.58
Arzati 2017, Zarezadeh 2018	23	100.54	7.69	22	101.86	11.96	-1.32 [-7.17, 4.53]	16.37
**Overall**							1.14 [-1.31, 3.58]	

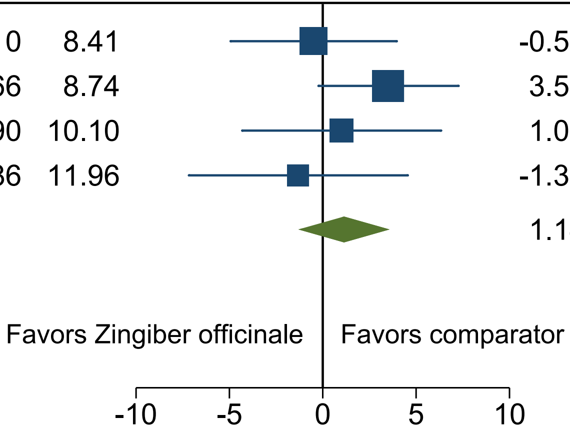

Heterogeneity: $\tau^2 = 0.57$, $I^2 = 9.01\%$, $H^2 = 1.10$

Test of $\theta_i = \theta_j$: $Q(3) = 2.75$, $p = 0.43$

Test of $\theta = 0$: $z = 0.91$, $p = 0.36$

Random-effects REML model
